# *Caryocar brasiliense* Camb. Fruit Improves Health and Lifespan in *Caenorhabditis elegans*

**DOI:** 10.1002/fsn3.70384

**Published:** 2025-06-07

**Authors:** Laura Costa Alves de Araújo, Natasha Rios Leite, Paola dos Santos da Rocha, Alex Santos Oliveira, Daniel Ferreira Leite, Isabella Giunco Estigarribia, Matheus Henrique Franco Alves, Alércio da Silva Soutilha, Helder Freitas dos Santos, Debora da Silva Baldivia, Nelson Carvalho Farias Junior, Danielle Araujo Agarrayua, Daiana Silva de Ávila, Jaqueline Ferreira Campos, Kely de Picoli Souza, Edson Lucas dos Santos

**Affiliations:** ^1^ Research Group on Biotechnology and Bioprospecting Applied to Metabolism and Cancer (GEBBAM) Federal University of Grande Dourados Dourados Mato Grosso do Sul Brazil; ^2^ Research Group in Biochemistry and Toxicology in Caenorhabditis elegans Federal University of Pampa Uruguaiana Rio Grande do Sul Brazil

**Keywords:** antioxidant, ascorbic acid, flavonoids, functional product, lycopene, neuroprotective, Pequi

## Abstract

The *Caryocar brasiliense* Camb. is a native Brazilian Cerrado species, considered the symbol of this biome. This tree produces a fruit known as pequi, which is much appreciated in Brazilian traditional culinary and is also used in popular medicine. However, there is still limited knowledge about the pharmaceutical potential of the fruit of *C. brasiliense* . In this study, we evaluated the bioactive compounds present in the lyophilized fruit pulp of *C. brasiliense* (CBFP) and investigated its antioxidant properties in vitro and in vivo, as well as its effects on models of Alzheimer's disease and longevity. As a main result, we revealed that CBFP presented phenolic and flavonoid constituents, lipophilic compounds, and ascorbic acid. The in vitro antioxidant activity was observed through free radical scavenging and DNA protection against oxidative damage. In the in vivo life quality assay, no signs of CBFP‐induced toxicity were observed, and nematode viability and reproductive capacity remained unaltered. Furthermore, CBFP treatment delayed paralysis in the Alzheimer's disease mutant strain and improved locomotor capacity during aging in the wild‐type strain. CBFP increased the lifespan of *C. elegans* and enhanced resistance to oxidative and heat stresses. Together, our findings demonstrate that CBFP exhibited beneficial effects on healthspan, attributed to its antioxidant properties and the regulation of oxidative stress.

## Graphical abstract

This study quantified bioactive compounds in *Caryocar brasiliense* fruit pulp and assessed its antioxidant capacity, heat and oxidative stress resistance, and quality of life improvements, including locomotor activity, resistance to β‐amyloid peptide toxicity, and pro‐longevity effects in *Caenorhabditis elegans* . The results show that *C. brasiliense* fruit pulp contains significant antioxidants with beneficial effects, including antioxidant, anti‐Alzheimer's, and healthspan properties in vivo.

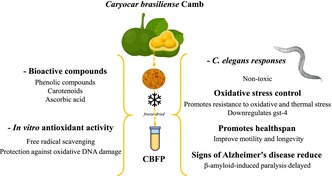

## 1 Introduction

Brazil hosts biomes rich in biodiversity, including the Cerrado, which represents a vast reservoir of natural resources. Within this biome, native fruit‐bearing species stand out for their unique sensory characteristics and high nutritional value. These native species have garnered significant attention, driving an increasing number of studies on their bioactive compounds and biological properties (Bailão et al. 2015; Borges et al. 2022).

Among this select group of plants, the species *Caryocar brasiliense* Camb., a member of the Caryocaraceae family, stands out for its prominence. This fruit species, which is considered a symbol of the Brazilian Cerrado, is edible, popularly known as pequi or pequiá, and widely used in the regional culinary field (Pinto et al. 2016). In traditional medicine, the fruit pulp has been used for treating stomach ailments and respiratory diseases (Roll et al. 2018). This species contains a diverse range of bioactive compounds already described, such as vitamin A, carotenoids, and phenolic compounds (Nascimento‐Silva and Naves 2019).

Biological and pharmacological activities have been primarily explored from the oil extracted from the pulp of *C. brasiliense* fruit, highlighting its chemoprotective potential against liver cancer (Palmeira et al. 2016), as well as its analgesic and anti‐inflammatory effects (Junior et al. 2019; Silva et al. 2023). Moreover, Traesel et al. (2016) demonstrated that the pulp fruit oil exhibits low acute and subchronic toxicity in animals. More recently, Fracasso et al. (2023) confirmed the anti‐inflammatory effect and the toxicological profile of the residue pulp fruit of the pequi. Furthermore, the flour obtained from the deseeded fruit peel contains phenolic compounds and anthocyanins and exhibits antioxidant activity (Leão et al. 2017).

The antioxidant compounds found in *C. brasiliense* are associated with beneficial cellular protection, counteracting reactive oxygen and nitrogen species, such as superoxide anion, hydroxyl radical, and hydroperoxyl, which play a key role in oxidative stress. Oxidative damage caused by these highly reactive molecules contributes to cellular senescence and is linked to the development and progression of neurodegenerative diseases, such as Alzheimer's disease (Jiang et al. 2016).

Fruits are considered important components of the human diet, and their consumption, combined with physical exercise, are strategies for improving quality of life and preventing diseases related to oxidative stress, such as neurodegenerative diseases (Miranda et al. 2017). Considering that dietary intervention and therapeutic strategies play crucial roles in improving quality of life and preventing diseases, it is essential to search for compounds or natural products that can act beneficially on mechanisms associated with oxidative and aging processes. However, studies on the biological and functional properties of pequi fruit pulp in vivo experimental models are scarce (da Costa Silva Kindelan et al. 2023; Fracasso et al. 2023).

Thus, this study aimed to characterize the bioactive compounds and evaluate the antioxidant capacity of *C. brasiliense* fruit pulp through in vitro and in vivo approaches. In vivo, the effects of the pulp were investigated in a *Caenorhabditis elegans* model, employing wild‐type and Alzheimer's disease mutant strains, to evaluate parameters related to the quality of life, including reproductive capacity, locomotor activity, resistance to toxicity from β‐amyloid peptide accumulation, and its impact on longevity.

## 2 Materials and Methods

### 2.1 Collect and Vegetal Material

Fruits of *C. brasiliense* , with a fresh weight of 2.6 kg, were collected in an area within the Cerrado biome, Dourados, Mato Grosso do Sul state, Brazil (S 21°59′ 41.8″ e W 55°19′ 24.9″), and transported with aeration at room temperature. A voucher specimen was identified and deposited in the Herbarium of the Federal University of Grande Dourados under the number 5412.

### 2.2 Preparation of *C. brasiliense* Fruit Pulp

To obtain the *C. brasiliense* fruit pulp (CBFP), fruits were washed under running water to remove impurities, sanitized by immersion in Sumaveg solution (3.3 g/L of water) for 15 min, rinsed with potable water, de‐pulped, lyophilized, and stored at −80°C.

For the experimental assay, 0.005 g of CBFP was resuspended in 5 mL of sterile Milli‐Q ultrapure water (1 mg/mL) and homogenized under constant agitation for 5 min. After this, we evaluated the higher dilution of the pulp and its chemical constituents. The resuspended CBFP was maintained under light protection and refrigeration at 4°C for 24 h. Just after this period, the CBFP was used for the analysis (Araújo et al. 2023).

### 2.3 Determination of the Phenolic and Flavonoid Compounds

For the determination of phenolic and flavonoid compounds, the CBFP was subjected to centrifugation at 5000 rpm for 10 min. The resulting supernatant was then collected and used for the subsequent analyses.

#### 2.3.1 Phenolic Compounds

The concentration of the phenolic compounds present on the CBFP was determined by the colorimetric method Folin–Ciocalteu, described by Meda et al. (2005). For this, Folin–Ciocalteu reagent (2.5 mL, 1:10 v/v, diluted in distilled water) was added to CBFP (0.5 mL at a concentration of 500 μg/mL). The solution was incubated for 5 min in the dark. Subsequently, 2 mL of 14% aqueous sodium carbonate (Na_2_CO_3_) was added and incubated at room temperature protected from light (120 min). The absorbance was measured at 760 nm using a T70 UV/Vis spectrophotometer (PG Instruments Limited, Leicestershire, UK). A calibration curve with gallic acid (0.0004–0.0217 mg/mL) was used as a standard. The phenolic compounds content present in CBFP was expressed in mg gallic acid equivalent (GAE)/g of pulp. Three independent assays were performed in triplicate.

#### 2.3.2 Flavonoids

The concentration of the flavonoids present in the CBFP was determined as described by Rocha et al. (2018). For this, a 2% ethanolic solution of aluminum chloride hexahydrate (AlCl_3_·6H_2_O) (4.5 mL) was added to 0.5 mL of CBFP (concentration of 500 μg/mL), and this solution was kept in the dark at room temperature for 30 min. Subsequently, the absorbance was measured at 415 nm (T70 UV/Vis spectrophotometer, PG Instruments Limited, Leicestershire, UK). The standard compound quercetin (0.0004–0.0217 mg/mL) was used to prepare the calibration curve. The total flavonoid content in CBFP was expressed as mg quercetin equivalent (QE)/g of pulp. Three independent assays were performed in triplicate.

#### 2.3.3 Determination of Lipophilic Compounds

The concentration of the lipophilic compounds present in the CBFP was determined as described by Rocha et al. (2019). For the determination of the lipophilic β‐carotene antioxidant compounds, lycopene and chlorophyll A and B were used: 150 mg of CBFP, vigorously homogenized in 10 mL of an acetone–hexane mixture (4:6, v/v) for 1 min, subsequently filtered using Whatman Grade 4 qualitative filter paper. The absorbances of the filtrate were measured at 453, 505, 645, and 663 nm. The β‐carotene, lycopene, and chlorophyll A and B contents were calculated according to mathematical equations: β‐carotene = 0.216 × Abs_663_–1.220 × Abs_645_–0.304 × Abs_505_ + 0.452 × Abs_453_; Lycopene = −0.0458 × Abs_663_ + 0.204 × Abs_645_ + 0.304 × Abs_505_–0.0452 × Abs_453_; Chlorophyll *a* = 0.9999 × Abs_663_–0.0989 × Abs_645_, and Chlorophyll *b* = −0.328 × Abs_663_ + 1.77 × Abs_645_. The results were expressed in mg/100 g of CBFP. Three independent assays were performed in triplicate.

#### 2.3.4 Ascorbic Acid Determination

The concentration of the ascorbic acid present in the CBFP was determined as described by Leite et al. (2020). To quantify the ascorbic acid concentration, CBFP (0.5 g) was vigorously homogenized in an oxalic acid solution (50 mL). Then, 20 mL of the solution was transferred to a 50 mL volumetric flask, and the volume was completed with oxalic acid. The mixture was filtered using Whatman Grade 4 qualitative filter paper. The filtrate was used to titrate a solution of the indicator DCFI (2,6‐Dichloroindophenol Sodium). The titration was terminated by the presence of a persistent pink color for 15 s. Ascorbic acid was used as a standard. The result was calculated based on the following equation and expressed in mg of ascorbic acid/100 g of CBFP:

$$\frac{M_{\mathrm{AA}}}{100\;g_{\text{sample}}}=\frac{\mathrm{DCF}I_{\text{sample}}}{\mathrm{DCF}I_{\text{standard}}}\times \frac{100}{M_{\text{sample}}}\times \frac{\left(M_{\text{solvent}}+M_{\text{sample}}\right)}{M_{\text{sample}}}\times \frac{50\;\mathrm{mL}}{10\;\mathrm{mL}}\times F$$

(1) $$F=\frac{M_{\mathrm{AA}}}{50}\times \frac{1}{25}\times 10$$

where DCFI_sample_ and DCFI_standard_ are respectively the volumes spent on the titration of the sample and standard in mL, *M*_sample_, *M*_solvent_, and *M*_pulp_ are respectively the mass quantity of sample, solvent added for sample titration, and sample aliquot (g). *F* is the amount of ascorbic acid required to reduce the DCFI (mg), and *M*_AA_ is the mass quantity of ascorbic acid (mg). Three independent experiments were performed in triplicate.

### 2.4 In Vitro Antioxidant Activity

#### 2.4.1 DPPH ^·^ Free Radical Scavenging

DPPH^·^ free radical scavenging assay was performed by the method described by Leite et al. (2020). For the experiment, 0.2 mL of CBFP (0.1–1000 μg/mL) was mixed with 1.8 mL of DPPH^·^ solution, 0.11 mM diluted in 70% ethanol. The mixture was homogenized and incubated at room temperature with protected from light, for 30 min. The absorbance was measured at 517 nm. Ascorbic acid and BHT (butylated hydroxytoluene) in concentrations of 0.1–1000 μg/mL were used as reference antioxidants. Three independent experiments were performed in triplicate. The inhibition curve was prepared, and the IC_50_ values (concentration required to inhibit 50% of the free radicals) were calculated. The percentage of DPPH^·^ free radical scavenging was calculated from the control (0.11 mM DPPH^·^ solution) using the following equation:

(2) $${\text{DPPH}}^{\cdot }\;\text{scavenging}\;\left(\%\right)=1-\frac{\mathrm{Abs}\;\text{sample}}{\mathrm{Abs}\;\text{control}}\times 100$$

#### 2.4.2 ABTS ^·+^ Free Radical Scavenging

ABTS^·+^ free radical scavenging assay was performed by the method described by Leite et al. (2020). For the assays, the ABTS^·+^ radical was prepared from a mixture of 5 mL of 2,2‐azino‐bis‐3‐ethylbenzothiazoline‐6‐sulfonic acid (ABTS) solution at a concentration of 7 mM and 88 μL of potassium persulfate solution (140 mM). The mixture was kept at room temperature for 12–16 h, protected from light. Then, the solution was diluted in absolute ethanol until an absorbance of 0.70 ± 0.05 at 734 nm was obtained. Posteriorly, 20 μL of CBFP (0.1–1500 μg/mL) was mixed with 1980 μL of the ABTS^·+^ radical. The solution was homogenized and incubated for 6 min at room temperature and protected from light. The absorbance was measured at 734 nm. Ascorbic acid and BHT were used as reference antioxidants (positive controls). Two independent assays were performed in triplicate. The inhibition curve was prepared, and the IC_50_ values were calculated. The percentage inhibition of ABTS^·+^ was determined according to the following equation:

(3) $${\text{ABTS}}^{\cdot +}\;\text{scavenging}\;\left(\%\right)=\left(\frac{\mathrm{Abs}\;\text{control}-\mathrm{Abs}\;\text{sample}}{\mathrm{Abs}\;\text{control}}\right)\times 100$$

#### 2.4.3 Oxidative Damage Induced to DNA

To evaluate the antioxidant capacity of CBFP against DNA damage, the plasmid‐induced oxidative damage assay was performed. The reaction mixture contained 4 μL phosphate‐buffered saline (PBS) and 50 ng/μL of pcDNA 3.1 plasmid + Gentamicin. For the assays, 4 μL of CBFP (1–2000 μg/mL), 4 μL of pcDNA 3.1 plasmid, and 4 μL of hydrogen peroxide (30%) were used. The samples were incubated in the transilluminator equipment (UVT‐312) at 302 nm at room temperature for 5 min. Posteriorly, the samples were transferred to the agarose gel (2%) containing ethidium bromide (10 mg/mL). The positive controls used were quercetin, catechin, gallic acid, and rutin. The gel was digitized in a Gel Doc EZ System photodocumenter and analyzed by Image Lab Software. The effects of CBFP were expressed as a percentage of fragmented DNA. Two independent assays were performed.

### 2.5 In Vivo Assays

#### 2.5.1 Strains and Maintenance Conditions of *C. elegans*

To perform the in vivo assays, wild‐type nematodes N2 Bistrol and the transgenic strains: CL2006 (dvIs2 [pCL12(unc‐54/human Aβ 1–42 minigene) + pRF4]); CF1553 muIs84 [(pAD76) sod‐3p::GFP + rol‐6(su1006)] and CL2166 dvIs19 [(pAF15) gst‐4p::GFP::NLS] III, obtained from the Caenorhabditis Genetics Center (CGC), Minnesota, USA, were used. The CL2006 strain has the Aβ 1–42 peptide that expresses the specific unc‐54 promoter gene, leading to progressive muscle paralysis in adult nematodes. For maintenance, the nematodes were kept in an incubator at 15°C or 20°C, cultured in Petri dishes containing *Nematode Growth Medium* (NGM), and fed with *Escherichia coli* (OP50) bacterium. The bacterium used as food for the nematodes was inactivated with the antibiotic Kanamycin (10 mM). To perform the assays, the nematode culture was synchronized with 2% sodium hypochlorite and 5 M sodium hydroxide following mechanical lysis.

In subchronic toxicity assays, the eggs resistant to alkaline and mechanical lysis were collected and transferred to Petri dishes containing NGM culture medium and *E. coli* (OP50) until they reached the L4 stage. After reaching the L4 development stage, these nematodes were transferred to microplates containing M9 buffer and subjected to different concentrations of CBFP in the absence of *E. coli* OP50.

For the other assays, eggs resistant to alkaline and mechanical lysis were collected and transferred to Petri dishes containing NGM culture medium, *E. coli* (OP50), and pretreated with water (Control) or concentrations of CBFP (400 or 1000 μg/mL) until they reached the L4 stage. For the assays of reproductive capacity, locomotor capacity, heat stress, and longevity, the nematodes continued to be maintained on plates containing solid NGM medium, *E. coli* OP50, and different concentrations of CBFP or water (Control). However, for the oxidative stress assays, SOD‐3 and GST‐4 expression, when they reached the L4 stage of development, the nematodes were transferred to M9 buffer in the absence of *E. coli* OP50, with different concentrations of CBFP.

#### 2.5.2 Subchronic Toxicity Assay

In this assay, we assessed the toxic effects of subchronic CBFP exposure in *Caenorhabditis elegans* (N2 strain). On average, 10 nematodes, synchronized in the L4 stage of development, were transferred to 96‐well microplates containing M9 buffer (100 μL) and CBFP (100 μL) at different concentrations (10 to 1000 μg/mL). Posteriorly, the nematodes were incubated at 20°C for 24 and 48 h. As a negative control, the nematodes were incubated only with M9 buffer (200 μL). After the incubation period, the viability of the nematodes was evaluated by touch sensitivity with the platinum wire. Three independent experiments were performed in triplicate.

#### 2.5.3 Reproductive Capacity Assay

To evaluate the effects of CBFP on the reproductive capacity of nematodes, we quantified the number of viable progeny during the five‐day reproductive period. In this assay, after synchronization, 5 nematodes at the L4 stage of development, pretreated with water (Control) or CBFP (400 or 1000 μg/mL), were transferred daily to new plates containing NGM/ *E. coli* (OP50) medium and water (Control) or CBFP at different experimental concentrations. The number of progenies was evaluated on each plate after reaching the L3 or L4 larval stage. The results are expressed as the average of three independent assays performed in triplicate.

#### 2.5.4 Locomotion Capacity Assay

The evaluation of the effect of CBFP on locomotor capacity was realized in two phases of the life cycle of N2 nematodes, comprising the first phase from egg to young adult (until day 2 in L4) and the second phase from egg to aging (until day 7 in L4). For this, after synchronization, on average, 10 nematodes (L4) pretreated with water (Control) or CBFP (400 or 1000 μg/mL) were daily transferred to new plates containing their respective treatments until they reached the first or second phase of the life cycle. After these periods, the nematodes were transferred to new plates containing only nematode growth medium (NGM), followed by acclimatization for 1 min and subsequent evaluation. In the evaluations, we quantified the number of sinusoidal curvatures during the 30 s of locomotion. Two independent assays were performed in triplicate.

#### 2.5.5 β‐Amyloid Induced Paralysis Assay

In this assay, we evaluated the effect of CBFP on progressive paralysis induced by β‐amyloid using CL2006 transgenic strains for Alzheimer's disease. Nematodes were synchronized (eggs) and exposed, or not, to CBFP for 72 h at 20°C until the L4 stage. Then, 20 nematodes per plate, in triplicate, were transferred to new plates containing *E. coli* with or without CBFP (400 or 1000 μg/mL). Posteriorly, to initiate β‐amyloid‐induced paralysis, nematodes were kept at 25°C for 22 h. After paralysis induction, evaluations were performed every 2 h for 10 h. The nematodes were classified as paralyzed in terms of their inability to move their bodies when stimulated by a platinum wire.

#### 2.5.6 Lifespan Assay

In the lifespan assay, N2 nematodes in the L4 phase, pretreated with water (Control) or CBFP (400 or 1000 μg/mL), were included. On the first day of the L4 adult phase (Day 1), 20 nematodes per group were transferred to new NGM/ *E. coli* OP50 plates containing their respective treatments. During the first 6 days, the reproduction period, the nematodes were transferred daily to new NGM plates containing their respective treatments. From the 7th day, the transfer to new plates occurred every 2 days. The evaluations consisted of classifying the nematodes as dead or alive until the day the death of the last nematode was recorded. The nematodes were considered dead when they did not move with or without stimulation by touch with a platinum wire. Nematodes with eggs hatched internally (bagging) or not visualized on the plates had their data excluded. Two independent assays were performed in triplicate.

#### 2.5.7 Heat Stress Assay

In the heat stress protection assay, an average of 20 nematodes (L4) pretreated with water (Control) or CBFP concentrations (400 or 1000 μg/mL) were transferred to new plates containing NGM/ *E. coli* (OP50) medium and water (Control) or CBFP (400 or 1000 μg/mL). Heat stress was induced by increasing the culture temperature from 20°C to 37°C and evaluated every hour of exposure for the experimental period of 6 h. The viability of nematodes exposed to 37°C in the different incubation periods was assessed after a 16 h recovery period at 20°C by touch sensitivity of a platinum wire. Three independent assays were performed in triplicate.

#### 2.5.8 Oxidative Stress Assay

The oxidative stress protection assay was performed by exposing nematodes to the oxidizing agent Juglone (5‐Hydroxy‐1,4‐naphthoquinone) at a lethal concentration (250 μM). Following synchronization, an average of 10 nematodes (L4), pretreated with water (Control) or CBFP (400 or 1000 μg/mL), were transferred to 96‐well microplates containing 100 μL of M9 buffer, 100 μL of CBFP (400 or 1000 μg/mL), and 50 μL of Juglone. As controls, nematodes were previously incubated with water and were exposed to 250 μL of M9 buffer (negative control) or 200 μL of M9 buffer plus 50 μL of Juglone (positive control). All microplates were incubated at 20°C, and nematode viability was evaluated every hour during the experimental period of 6 h. The viability of the nematode was assessed by touch sensitivity using a platinum wire. Three independent assays were performed in triplicate.

#### 2.5.9 Expression of SOD‐3 and GST‐4 Marked With GFP

To analyze the expression of the antioxidant enzymes superoxide dismutase (SOD‐3) and glutathione transferase (GST‐4), the GFP‐marked strains CF1553 and CL2166, respectively, were used. After synchronization, 5 nematodes (L4) pretreated with water (Control) or concentrations of CBFP (400 or 1000 μg/mL) were immediately transferred to staining slides containing 1 mM levamisole as an anesthetic. The images were captured using an epifluorescence microscope (Nikon Eclipse 50i) connected to a digital camera (Samsung ST64). Images of 5 nematodes per group were expressed as pixel averages, and the relative fluorescence of the whole body was determined using ImageJ software. Three independent assays were performed in triplicate.

### 2.6 Statistical Analysis

Statistical analyses were performed using GraphPad Prism 5.1 software (San Diego, CA, USA). Data are expressed as the mean ± standard error of the mean (SEM). Significant differences between groups were determined using Student's *t*‐test for comparison between two groups and analysis of variance (ANOVA), followed by *Dunnett's* test for comparison of three or more groups. For life expectancy assays, the Kaplan–Meier curve was used, and *p* values were calculated by the Log‐rank test. Results were considered significant when *p* < 0.05.

## 3 Results

### 3.1 Yield and Identification of Bioactive Compounds From CBFP

The yield obtained from the *in natura* pulp after the freeze‐drying process was 43.73%. The concentrations of the bioactive compounds present in the CBFP are presented in Table 1.

**TABLE 1:** Bioactive compound present on the pulp fruit *C. brasiliense* (CBFP).

Sample	Phenolic compounds	Flavonoids	*β*‐carotene	Lycopene	Chlorophyll *a*	Chlorophyll *b*	Ascorbic acid
	mg de AGE/100 g	mg de QE/100 g	mg/g	mg/g	μg/g	μg/g	mg/100 g
CBFP	572.25 ± 0.14	160.80 ± 0.09	N.D.	1.87 ± 0.19	3.29 ± 0.349	5.52 ± 0.58	600.22 ± 6.54

*Note:* Results are expressed as mean ± SEM.

Abbreviation: N.D., not detected.

### 3.2 Antioxidant Activity In Vitro of CBFP

The in vitro antioxidant activity of CBFP, expressed as the concentration required to inhibit 50% (IC_50_) of the DPPH^·^ and ABTS^·+^ radicals, is presented in Table 2. CBFP was more efficient in scavenging the ABTS^·+^ radical compared to the DPPH^·^ radical, showing an IC_50_ approximately 0.38 times lower.

**TABLE 2:** Antioxidant activity of *C. brasiliense* pulp fruit (CBFP).

Samples	DPPH^·^	ABTS^·+^
	IC_50_ (μg/mL)	IC_50_ (μg/mL)
Ascorbic acid	2.65 ± 0.20	1.43 ± 0.09
BHT	14.58 ± 2.15	10.15 ± 0.94
CBFP	394.70 ± 25.9	245.35 ± 4.95

*Note:* The results are expressed as mean ± SEM.

### 3.3 CBFP Protects Against Oxidative Damage to DNA

**FIGURE 1:**
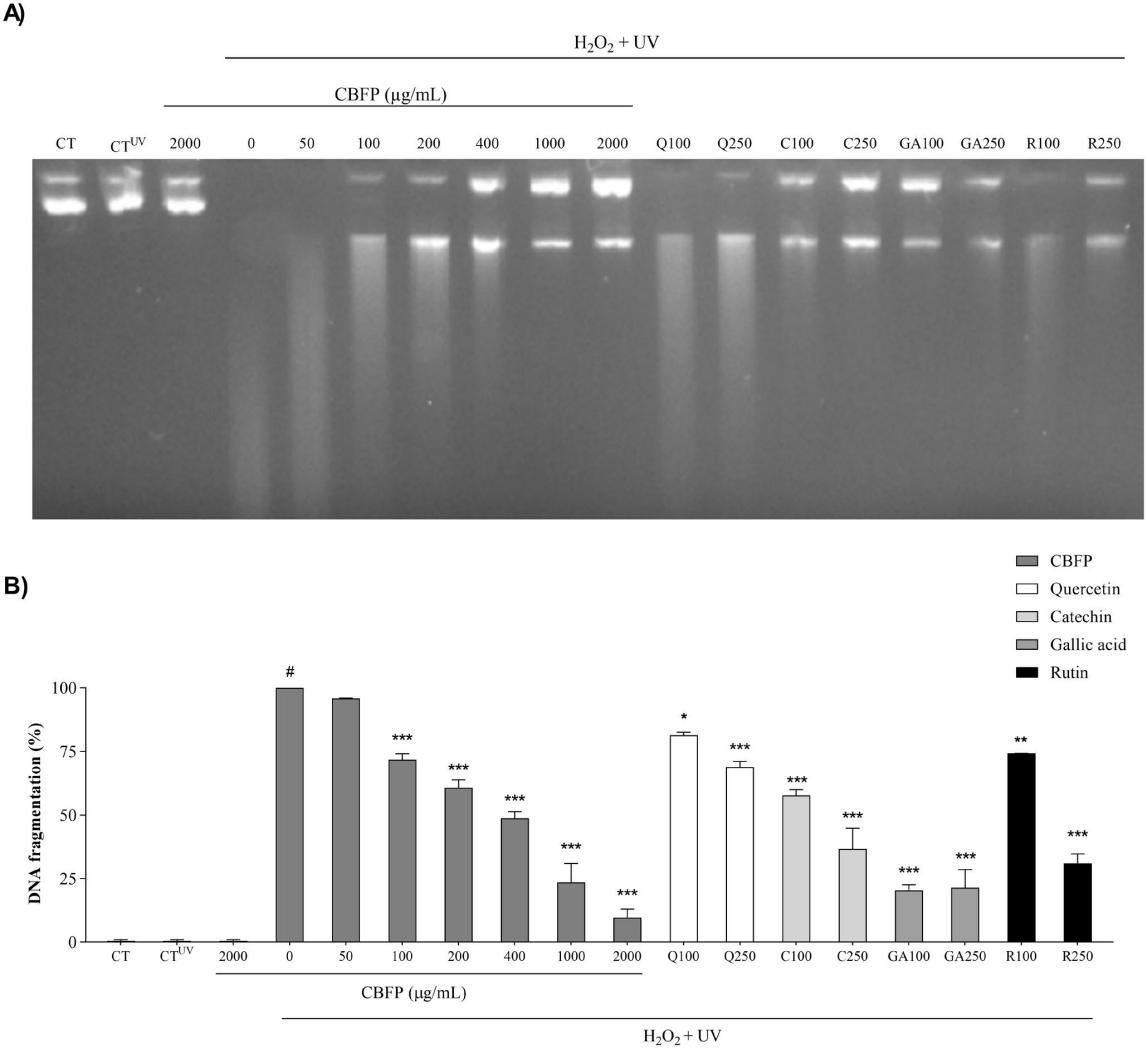
DNA protection by *C. brasiliense* fruit pulp (CBFP): (A) representative image of pcDNA3.1 in 2% agarose gel; (B) percentage of fragmented DNA exposed to damage with H_2_O_2_ + UV and, treated with CBFP (50–2000 μg/mL), and controls (Q = Quercetin C = Catechin; GA = Gallic Acid; R = Rutin 100 and 250 μg/mL). CT = plasmid DNA; CTUV = plasmid DNA + UV. Values are expressed as mean ± SEM. #*p* < 0.05 versus CT. *p < 0.05, **p < 0.01 and ***p < 0.001 versus CBFP 0 μg/mL.

In Figure 1, treatment with the oxidizing reagent H_2_O_2_ and UV radiation completely damaged the DNA, as can be observed by the disappearance of the DNA band in the 0 μg/mL CBFP group, compared with the CT group. We observed that CBFP exhibited a dose‐dependent protective capacity against the induction of DNA damage from 100 μg/mL of CBFP. This protection, demonstrated by the progressive restoration of the integrity of the DNA band, was similar to the effects of the control antioxidants quercetin, catechin, gallic acid, and rutin.

### 3.4 In Vivo Assays

#### 3.4.1 CBFP Does Not Promote Toxic Effects in *C. elegans*

**FIGURE 2:**
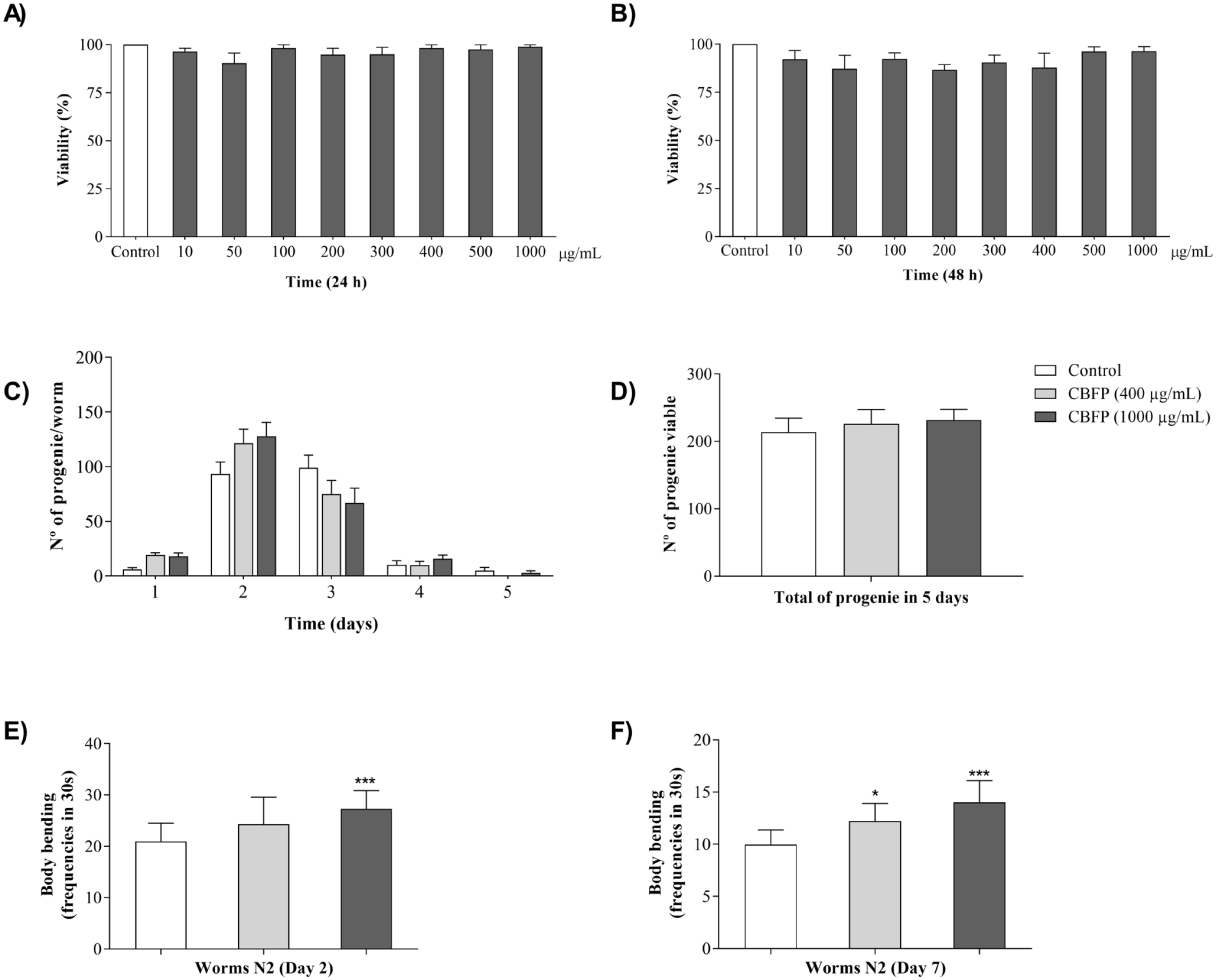
Effects of *C. brasiliense* fruit pulp (CBFP) on viability and healthspan promotion in *C. elegans*: (A) viability after 24 h of treatment; (B) viability after 48 h of treatment; (C) daily quantification of the number of progenies; (D) total quantification of the number of progenies during 5 days; (E) locomotion capacity in the young adult stage and (F) locomotion capacity in the aging stage. Values are expressed as mean ± SEM. *p < 0.05 and ***p < 0.001 when the treated group was compared with the Control group.

In this assay, we evaluated the toxicity of CBFP in vivo, in nematodes exposed to subchronic treatment conditions. In Figure 2A,B, no toxic effects were observed at concentrations between 10 and 1000 μg/mL after 24 and 48 h of treatment. From these results, we defined the safe concentrations for the next assays.

#### 3.4.2 CBFP Promotes Healthspan in *C. elegans*

To investigate the effects of CBFP on quality of life, we evaluated the effects on indicators that represent the healthy physiological pattern, including reproductive capacity, locomotor capacity, and longevity in *C. elegans* .

In the evaluations of the effects of CBFP on the number of viable progeny of nematodes, we observed that the evaluated concentrations of CBFP did not promote changes in the daily or total number of viable progeny (Figure 2C,D). These results indicate that treatments with different concentrations of CBFP do not promote toxic effects that interfere with the physiological patterns of nematode reproductive capacity.

As nematodes age, their motility progressively decreases; therefore, we analyzed locomotion at different life cycle stages, including the aging phase. We observed that treatment with 1000 μg/mL of CBFP resulted in approximately a 30% increase in motility during the young adult phase compared to control nematodes (Figure 2E). The beneficial effects on the motility of treated nematodes were more pronounced during the aging phase (Figure 2F). In this phase, both doses of CBFP, 400 and 1000 μg/mL, increased the number of body curvatures by 22.61% and 40.70% compared to the Control group, respectively.

**FIGURE 3:**
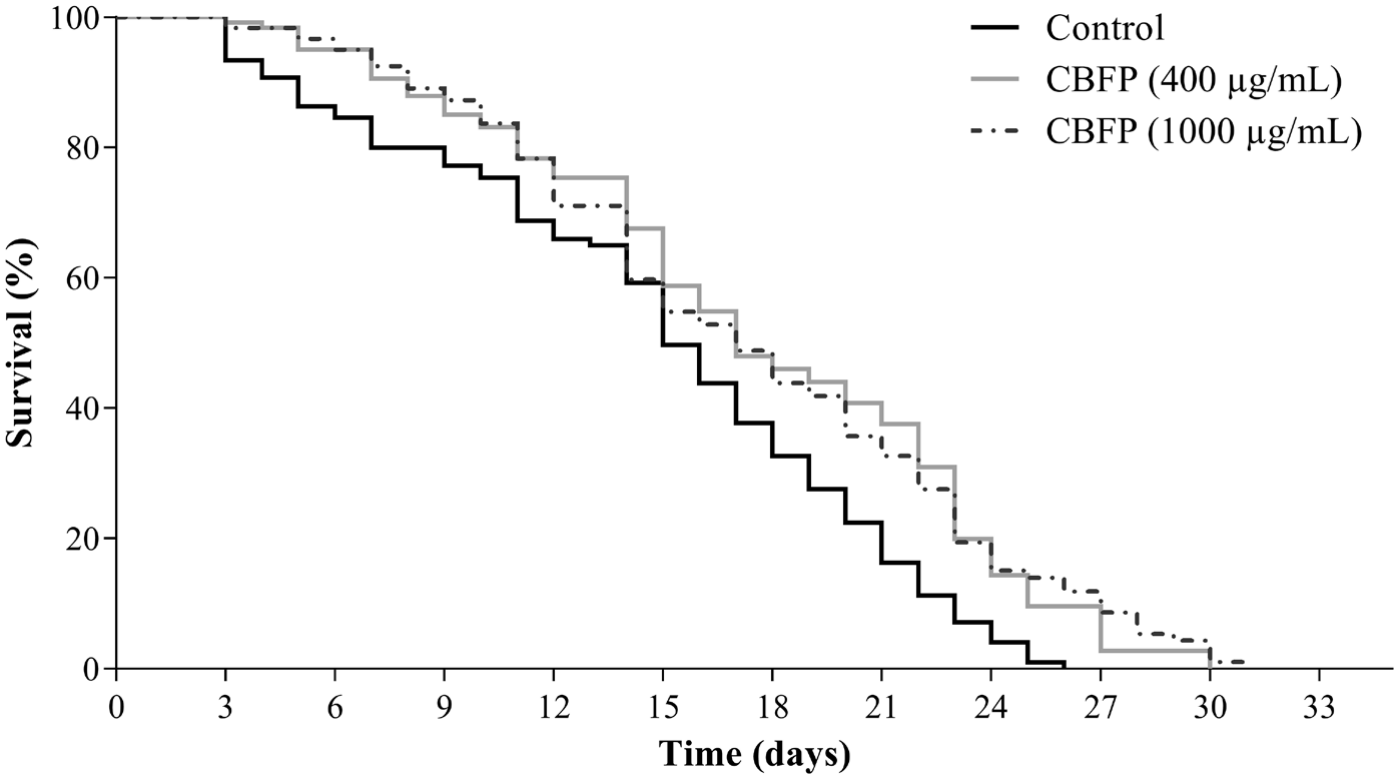
Effects of *C. brasiliense* fruit pulp (CBFP) on the longevity of *C. elegans*.

We evaluated the effects of CBFP on the life expectancy of nematodes, observing that nematodes in the Control group lived an average time of 16 ± 1.0 days (maximum of 26 days), while those treated with CBFP 400 or 1000 μg/mL had an average lifespan of 17 ± 2.0 days (maximum of 30 days) and 17 ± 3.0 days (maximum of 31 days), respectively (Figure 3). According to the results, the CBFP concentrations evaluated exhibited similar survival profiles, with a significant increase corresponding to 6.25% in the average lifespan for both concentrations, compared to the Control group (Table S1). Already in the maximum lifespan time, the increase was approximately 8% and 20% for nematodes treated with 400 and 1000 μg/mL of CBFP, respectively, compared to the Control group (Figure 3).

#### 3.4.3 CBFP Delays and Paralysis Caused by β‐Amyloid (Aβ) Expression

**FIGURE 4:**
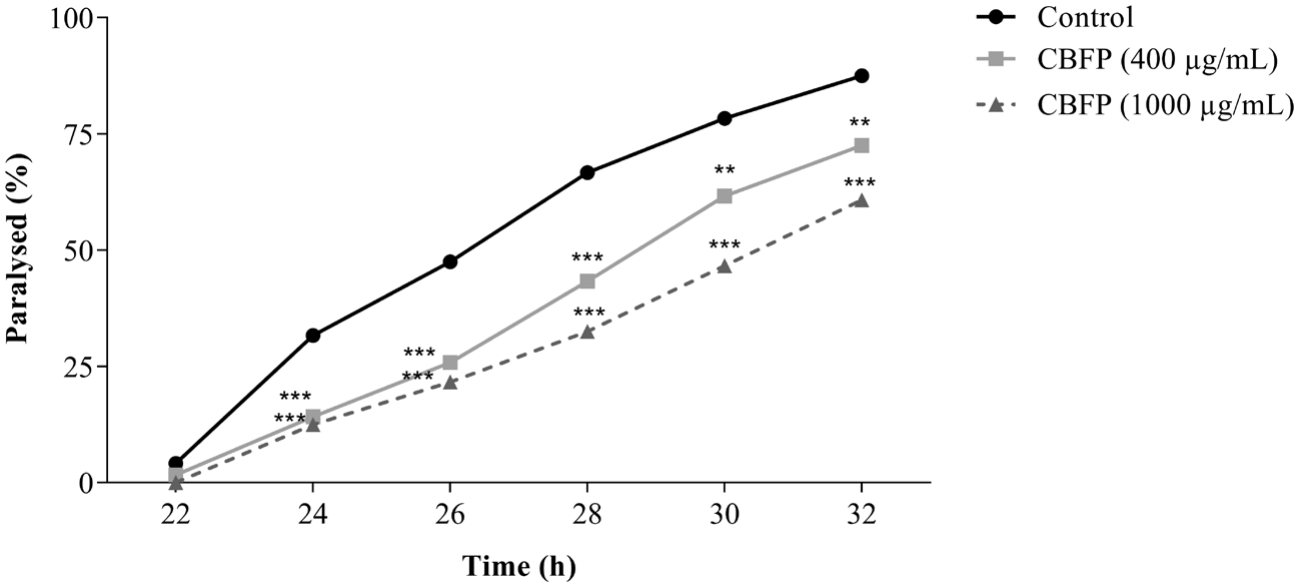
Paralysis curve induced in *C. elegans* (CL2006) treated with *C. brasiliense* fruit pulp (CBFP). Values are expressed as mean ± SEM. **p < 0.01 and ***p < 0.001 versus control.

Alzheimer's disease mutant nematodes treated with CBFP exhibited positive alterations in parameters indicative of β‐amyloid toxicity. The results of the effects of CBFP on paralysis are presented in Figure 4 and demonstrate that CBFP treatments promote significant delays in β‐amyloid‐induced paralysis in 27.5% of the treated nematodes (400 μg/mL) and 39.17% when treated at the highest concentration evaluated (1000 μg/mL). These results indicate that CBFP treatments minimize the toxicity caused by the β‐amyloid peptide.

#### 3.4.4 CBFP Promotes Protection Against Heat and Oxidative Stress

**FIGURE 5:**
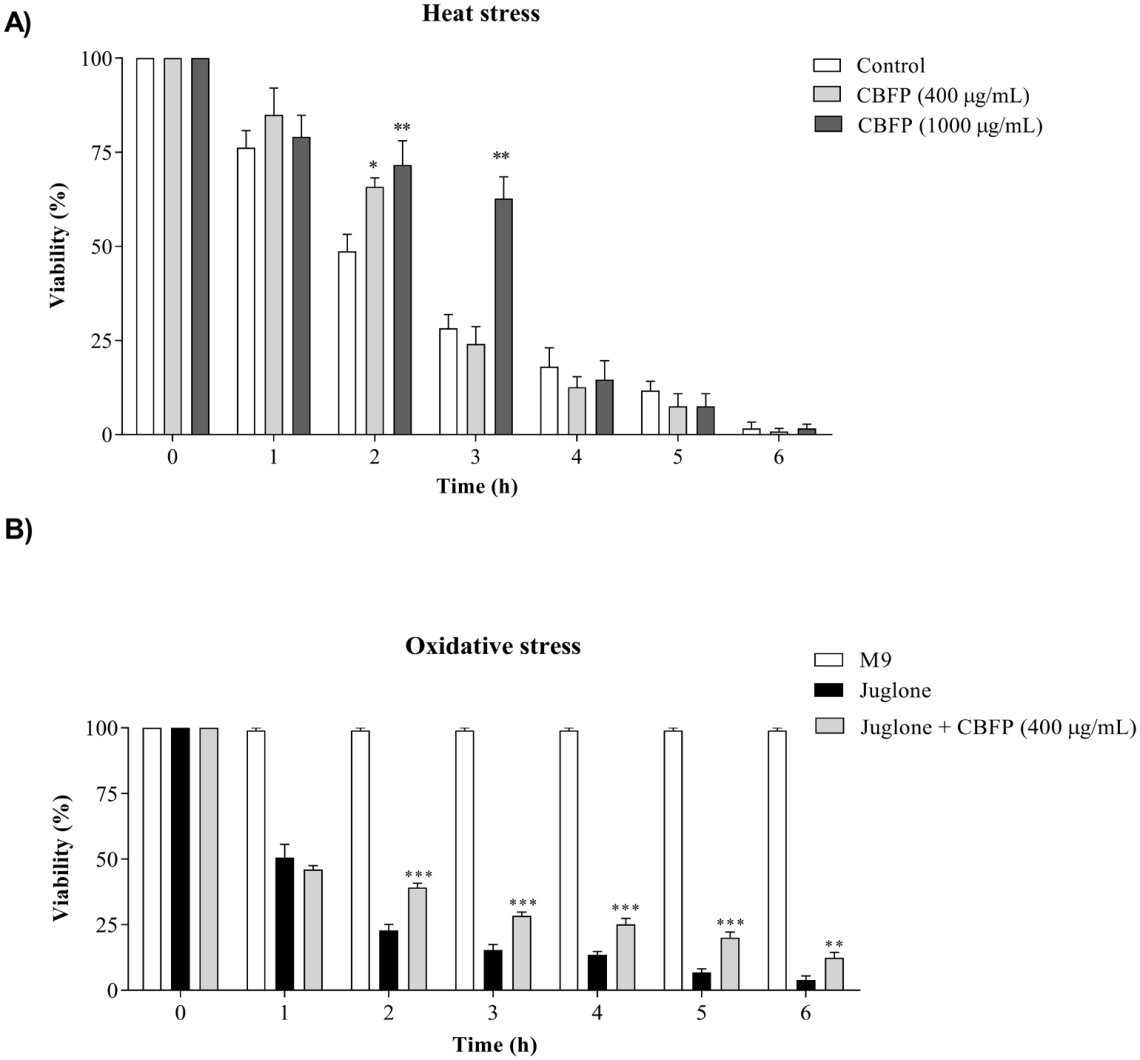
Effects of *C. brasiliense* fruit pulp (CBFP) on heat stress and oxidative stress in *C. elegans*: (A) exposed to 37°C for 6 h; (B) subjected to the pro‐oxidant reagent Juglone for 6 h. Values are expressed as mean ± SEM. *p < 0.05 and **p < 0.01 versus control.

The effects of CBFP on nematodes exposed to adverse heat and oxidative stress conditions are shown in Figure 5. The protective effect against heat stress was observed in nematodes treated with CBFP in the second and third hours of evaluation (Figure 5A). In the second hour, the percentage of viable nematodes was 48.7% for the Control group, and 65.8% and 71.6% for the groups treated with 400 and 1000 μg/mL of CBFP, respectively. In the third hour of evaluation, the concentration of 1000 μg/mL maintained 62.8% of the viable nematodes, while in the Control group, only 28.0% of the nematodes were viable. However, CBFP was not able to protect the nematodes from long periods of exposure to heat stress.

To the protective effect against oxidative stress, we observed that the concentration of 400 μg/mL of CBFP promoted a significant protective effect from the second hour of exposure (Figure 5B). In this assay, it was not possible to observe the effect of the treatment at the concentration of 1000 μg/mL due to the intense coloration obtained from the Juglone + CBFP solution (1000 μg/mL). We observed that the lowest concentration evaluated (400 μg/mL) exhibited a protective effect during the period corresponding to 2 h. The viability of the nematodes ranged from 100% to 3.91% in the control group and from 100% to 12.38% in the group treated with CBFP 400 μg/mL in the evaluated period of 6 h. The viability of the animals treated with CFP was between 2 and 3 times higher than that of the control animals at the times evaluated. The percentages of viable nematodes are presented in Table S2.

#### 3.4.5 CBFP Regulates the Expression of Antioxidant Genes

**FIGURE 6:**
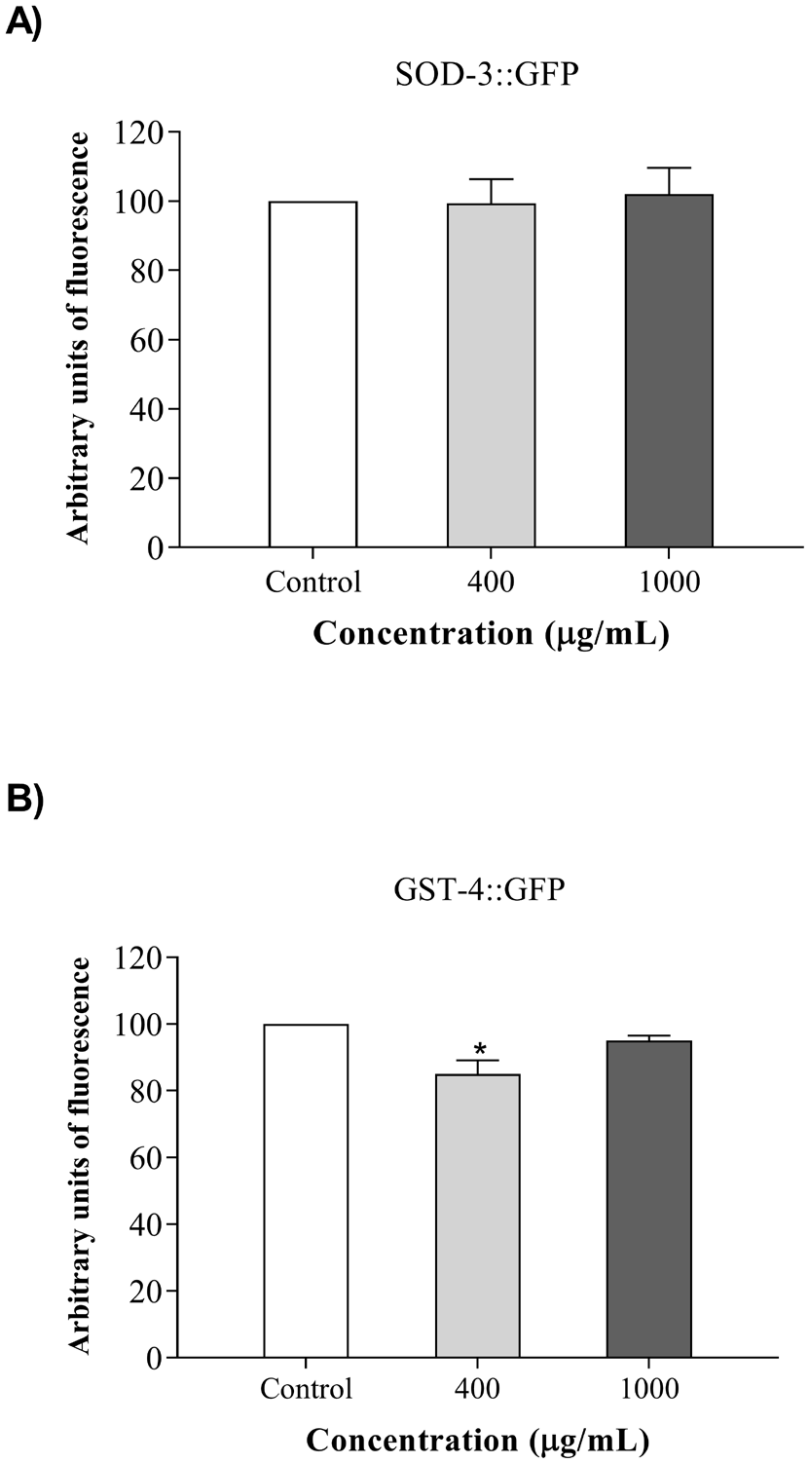
Expression of the antioxidant gene on *C. elegans* treated with the pulp fruit of *C. brasiliense* (CBFP): (A) expression of SOD‐3::GFP (CF1553 [sod‐3p:GFP]); (B) expression of GST‐4::GFP (CL 2166 [gst‐4p:GFP]). The values are expressed as mean ± SEM. *p < 0.05 versus control.

To demonstrate the effects on the modulation of target genes related to the antioxidant system, we used the transgenic strains CF1553 (SOD‐3::GFP) and CL2166 (GST‐4::GFP). We observed in Figure 6A that there was no increase in the expression of SOD‐3 in CF1553 strains treated with CBFP (400 or 1000 μg/mL), when compared with the Control group, indicating that the basal conditions were maintained. In the CL2166 (GST‐4::GFP) strains treated with CBFP (400 μg/mL), the expression of GST‐4 was negatively regulated (Figure 6B).

## 4 Discussion

Several fruit species from Brazilian biodiversity demonstrate potential for developing new functional foods and nutraceuticals with active ingredients of therapeutic importance and reduced toxicity.

This study utilized the freeze‐dried pulp of the Brazilian native fruit *C. brasiliense* and identified relevant preserved bioactive compounds. The in vitro antioxidant activity of CBFP was demonstrated through free radical scavenging methods. We associate this antioxidant activity with phytochemical constituents identified in CBFP, such as phenolic compounds (flavonoids), carotenoids (lycopene), and ascorbic acid. These compounds have been described in other plant parts of the same species, including phenolic compounds in the fruit peels (Brito Cangussu et al. 2021; Caldeira et al. 2021; Melo et al. 2024) and carotenoids in the oil and flour from the pulp of fruits and almonds (Narita et al. 2022; Brito et al. 2022; Carneiro et al. 2023) and ascorbic acid in the fruit pulp (Machado et al. 2013). Furthermore, it has also been reported in other species of the genus *Caryocar*, such as phenolic compounds, carotenoids, and ascorbic acid in the pulp of the fruits of the species *C. villosum* (Barreto et al. 2009; Chisté and Mercadante 2012b) and phenolic compounds in the pulp and peel of the fruit *C. coriaceum* (Alves et al. 2017).

In the literature, it is well elucidated that the compounds identified in CBFP are antioxidants. Almeida‐Bezerra et al. (2022) described in a review article the antioxidant potential of the leaves, fruit peel, and pulp of *C. coriaceum*. In studies by Chisté et al. (2012a), the antioxidant potential of *C. villosum* fruit pulp was reported. Melo et al. (2024) and Machado et al. (2013) described the in vitro antioxidant potential of the fruit peel and pulp of the species under study, *C. brasiliense* . Among the mechanisms of action of antioxidant compounds, free radical scavenging is well described due to the presence of free hydroxyl groups in their structures, which are capable of donating electrons and/or hydrogen atoms to other unstable and reactive molecules, such as free radicals, and thus reducing oxidative damage in biomolecules (Rudrapal et al. 2024) in oxidative stress condition. Continuous exposure to reactive species promotes oxidation of lipids and proteins in cell membranes, as well as oxidative DNA damage. This way, the accumulation of DNA damage can trigger mutagenesis processes, which have been associated with oncogenesis, premature aging, and the emergence of age‐related diseases, including metabolic diseases and Alzheimer's disease (Kwiatkowski et al. 2016; Shimizu et al. 2014).

In this study, we examined the effects of the antioxidant activity of CBFP on DNA under conditions of induced oxidative stress. CBFP protects DNA from oxidative damage in a dose‐dependent manner. This protection can be attributed to direct scavenging mechanisms of hydroxyl radicals generated by the photolysis of hydrogen peroxide under ultraviolet light (Kapoor and Dharmesh 2016). In vivo, other mechanisms of action are also described, such as indirect mechanisms through modulation of endogenous antioxidant systems, including enzymatic pathways that prevent oxidative damage (Davalli et al. 2018).

This study reports for the first time the antioxidant properties of the freeze‐dried pulp of the fruit of *C. brasiliense* in vivo in the biological model *C. elegans* . Initially, subchronic exposure to CBFP at different concentrations revealed no toxic effects on the viability of the treated nematodes, providing the safety of use necessary for studies of potential pharmacological activities.

Nascimento‐Silva and Naves (2019) described the species *C. brasiliense* as a medicinal food, and several beneficial effects on health were reported, including analgesic, anti‐inflammatory, and antioxidant actions. However, studies on the neuroprotective effects of *C. brasiliense* against neurodegenerative diseases, such as Alzheimer's disease, are still scarce. In the study by De Oliveira et al. (2018), it was demonstrated that leaf extracts of *C. brasiliense* exhibited anticholinesterase and antioxidant properties. Based on these pharmacological potentials, we demonstrated that CBFP exerts beneficial effects in a mutant strain of *C. elegans* used as a model for Alzheimer's disease, including the delay of β‐amyloid‐induced paralysis. In a wild‐type strain of *C. elegans* , CBFP improved locomotor activity at different life cycle stages, without altering the number of viable progenies in *C. elegans* . Compared with the Control group, CBFP significantly inhibited the decline in locomotor capacity that occurred with age, especially during the aging phase. In this context, we demonstrated that the decline in age‐related functions, including reproductive capacity and locomotion, was significantly delayed in the treated nematodes. This indicates that CBFP could also enhance the quality of life in aged nematodes. Furthermore, CBFP promoted an increase in longevity observed by extending the average and maximum life expectancy of treated nematodes.

Increased life expectancy is related to better conditions for regulating oxidative and heat stresses, which reduce quality of life and longevity (Vatner et al. 2020). The antioxidant capacity of CBFP reduced the impacts of stress conditions induced by high temperature or pro‐oxidant agents in vivo. We observed that CBFP promotes protective effects in *C. elegans* exposed to high temperature for 3 h. Still, under oxidative stress conditions, CBFP demonstrated a better protective effect, being effective throughout the evaluated period. A similar effect was shown by Roxo et al. (2020) when investigating the antioxidant activity of *C. villosum* fruits in *C. elegans* . The authors attributed the effect of antioxidant activity to mechanisms of free radical scavenging and regulation of the expression of genes involved in defense signaling pathways.

In *C. elegans* , the Daf‐16/IGF‐1 (IIS), SKN‐1/Nrf, and HSF‐1/HSF pathways modulate the expression of genes involved in responses to heat and oxidative stress and contribute to increased lifespan and longevity, mechanisms that are highly conserved in mammals, including humans (Blackwell et al. 2015; Kumsta et al. 2017; Sun et al. 2017). DAF‐16 is a key transcription factor for longevity, and its nuclear localization is essential for the transcriptional activation of a wide array of target genes, including the antioxidant enzymes CTL‐1 and SOD‐3. Under stress conditions, the transcription factor SKN‐1 positively regulates the expression of Phase II detoxification genes, including GCS‐1 and GST‐4. Under normal conditions, SKN‐1 associates with the WDR‐23 protein and the CUL‐4/DDB ubiquitin ligase protein complex, promoting its degradation by the proteasomal system (Tang and Choe 2015). However, it has also been described that nuclear and cytoplasmic isoforms of WDR‐23 mediate differential effects on SKN‐1 and consequently modulate the negative regulation of Phase II detoxification gene expression (Spatola et al. 2019).

Thus, to understand possible underlying mechanisms by which CBFP exerts beneficial effects in *C. elegans* , we evaluated the expression levels of SOD‐3 and GST‐4 genes, related to the antioxidant defense mechanism. Our study revealed that CBFP did not alter the expression of SOD‐3 and negatively regulated the expression of GST‐4. The negative regulation of GST‐4 and increased lifespan by CBFP corroborate data found by Roxo et al. (2020) for *C. elegans* treated with extract from the peels of *C. villosum* fruits. This type of retrograde response is known as mitochondrial hormesis and may have potential applications for health‐promoting effects in humans (Ristow and Zarse 2010).

Thus, we can suggest that by downregulating GST‐4, there is an increase in the formation of mitochondrial reactive oxygen species, promoting an adaptive response that results in a subsequent increase in resistance to stress and an increase in the life expectancy of *C. elegans*. These adaptive responses to cellular stress, called hormetic pathways, can also be activated by phytochemicals, including the activation of different kinases and transcriptional factors that induce the expression of genes encoding antioxidant enzymes, chaperone proteins, neurotrophic factors, and other cytoprotective proteins that promote health and longevity (Calabrese et al. 2011; Martel et al. 2019).

In conclusion, this study demonstrates that the pulp of *Caryocar brasiliense* fruits contains significant antioxidant compounds. These compounds showed beneficial antioxidant, anti‐Alzheimer's, and healthspan‐enhancing effects in the in vivo *C. elegans* model. Although the data obtained is significant, future HPLC analyses may be conducted to pinpoint the compounds responsible for the observed activities. These findings suggest potential applications for health promotion and the prevention of diseases associated with oxidative stress.

## Author Contributions

**Laura Costa Alves de Araújo:** conceptualization (equal), data curation (equal), formal analysis (equal), investigation (lead), methodology (equal), validation (lead), visualization (equal), writing – original draft (equal), writing – review and editing (equal). **Natasha Rios Leite:** conceptualization (equal), data curation (equal), formal analysis (equal), investigation (equal), methodology (equal), validation (equal), visualization (equal), writing – original draft (equal), writing – review and editing (equal). **Paola dos Santos da Rocha:** conceptualization (equal), formal analysis (equal), methodology (equal), validation (equal), visualization (equal), writing – original draft (equal), writing – review and editing (equal). **Alex Santos Oliveira:** conceptualization (equal), methodology (equal), visualization (equal), writing – original draft (equal), writing – review and editing (equal). **Daniel Ferreira Leite:** conceptualization (equal), methodology (equal), visualization (equal), writing – original draft (equal), writing – review and editing (equal). **Isabella Giunco Estigarribia:** conceptualization (supporting), methodology (supporting), visualization (supporting), writing – review and editing (equal). **Matheus Henrique Franco Alves:** conceptualization (supporting), methodology (supporting), visualization (supporting), writing – review and editing (supporting). **Alércio da Silva Soutilha:** conceptualization (supporting), methodology (supporting), visualization (supporting), writing – review and editing (equal). **Helder Freitas dos Santos:** conceptualization (equal), methodology (equal), visualization (equal), writing – original draft (equal), writing – review and editing (equal). **Debora da Silva Baldivia:** conceptualization (equal), methodology (equal), visualization (equal), writing – original draft (equal), writing – review and editing (equal). **Nelson Carvalho Farias Junior:** methodology (equal), visualization (equal), writing – review and editing (equal). **Danielle Araujo Agarrayua:** methodology (equal), visualization (equal), writing – review and editing (equal). **Daiana Silva de Ávila:** conceptualization (equal), data curation (equal), methodology (equal), resources (equal), supervision (equal), visualization (equal), writing – review and editing (equal). **Jaqueline Ferreira Campos:** conceptualization (equal), supervision (equal), visualization (equal), writing – review and editing (equal). **Kely de Picoli Souza:** conceptualization (equal), resources (equal), supervision (equal), visualization (equal), writing – original draft (equal), writing – review and editing (equal). **Edson Lucas dos Santos:** conceptualization (lead), data curation (lead), funding acquisition (lead), methodology (equal), project administration (lead), resources (lead), supervision (lead), visualization (equal), writing – original draft (equal), writing – review and editing (lead).

## Conflicts of Interest

The authors declare no conflicts of interest.

## Supporting information


Table S1

## Data Availability

The data that support the findings of this study are available on request from the corresponding author.
