# Supplementary material for: Caryocar brasiliense Camb. Fruit Improves Health and Lifespan in Caenorhabditis elegans
Source: Food Sci Nutr. 2025 Jun 7;13(6):e70384. doi: 10.1002/fsn3.70384 (PMC12144583; doi:10.1002/fsn3.70384)
Supplement: Supplementary file 1 — Table S1 [file FSN3-13-e70384-s001.docx]

Table S1. Average lifespan in *C. elegans* control and treated with CBFP.

| Treatment  (µg/mL) | Mean lifespan (Days) | Extension da mean lifespan  (%) | Log–rank Test vs. Control | Number of worms |
| --- | --- | --- | --- | --- |
| Control | 15 ± 1.0 | - | - | 120 |
| CBFP 400 | 17.0 ± 2.0 | 13.33 | <0.001*** | 120 |
| CBFP 1000 | 17.0 ± 3.0 | 13.33 | <0.01** | 120 |

Values ​​are expressed as mean ± SEM. **P<0.01 and ***P<0.001 versus Control.

Table S2. Viability analysis of *C. elegans* exposed to oxidative stress.

| Hour | Juglone  *(pro-oxidant)* | | Juglone + CBFP (400 µg/mL)  *(pro-oxidant + antioxidant)* | |
| --- | --- | --- | --- | --- |
|  | % Viability | SEM | % Viability | SEM |
| 0 | 100.00 | 0.00 | 100.00 | 0.00 |
| 1 | 50.47 | 5.14 | 45.99 | 1.52 |
| 2 | 22.76 | 2.34 | 39.13 | 1.64 |
| 3 | 15.34 | 2.11 | 28.37 | 1.42 |
| 4 | 13.46 | 1.26 | 25.12 | 2.21 |
| 5 | 6.80 | 1.35 | 20.01 | 2.16 |
| 6 | 3.91 | 1.59 | 12.38 | 2.05 |
